# Knowledge of and Adherence to Hygiene Guidelines among Medical Students in Austria

**DOI:** 10.1155/2013/802930

**Published:** 2013-04-11

**Authors:** Verena G. Herbert, Paul Schlumm, Harald H. Kessler, Andreas Frings

**Affiliations:** ^1^Dermatologikum Hamburg, 20354 Hamburg, Germany; ^2^Medical University of Graz, 8036 Graz, Austria; ^3^Institute for Hygiene, Microbiology and Environmental Medicine, Medical University of Graz, 8010 Graz, Austria; ^4^Department of Ophthalmology, University Medical Center Hamburg-Eppendorf, 20246 Hamburg, Germany

## Abstract

*Background*. Adherence to hygiene guidelines is of utmost importance for healthcare professionals. The aim of this study was to evaluate the knowledge on and the adherence to hygiene guidelines among medical students in Austria. Additionally, a possible difference between female and male students was investigated. *Methods*. An open paper-based survey among third-year medical students at the Medical University of Graz was conducted. The questionnaire consisted of 20 single-choice questions covering compliance with basic hygiene standards, self-rated knowledge of hygiene guidelines, and satisfaction with current hygiene education, equipment, and quality standards. *Results*. Of 192 medical students, 70% judged their knowledge of hygiene standards as “excellent” or “good”; however, only 49% reported adherence to hygiene guidelines and only 43% performed hygienic hand disinfection according to WHO guidelines. Of the respondents, 79% voted for a mandatory course on hygiene standards in medical education. No significant gender differences were observed. *Conclusion*. While the knowledge on hygiene guidelines appears to be good among medical students, adherence is limited and requires improvement. The need for an optimum education in hygiene is high.

## 1. Introduction

Healthcare-associated infections pose a continuing threat for mortality and morbidity among hospitalized patients [1]. Hospital-acquired infections mainly draw attention because of the growing awareness that most of them are preventable [2]. Evidence suggests that proper hand hygiene practice is regarded as the single most effective and simple inexpensive strategy for reducing the prevalence of hospital-acquired infections [1, 3–7]. However, adherence to good hand hygiene practice remains consistently poor in the clinical setting [7]. The hygiene adherence by healthcare professionals has been described previously [2, 7], whereas compliance of medical students has rarely been examined [8–11].

Assessment and raising awareness of hygiene standards during undergraduate education may affect the behavior of graduate students upon entering professional life and contribute to the reduction of nosocomial infection rates. Thus, the present study was performed to examine the knowledge on and the adherence to hygiene guidelines among medical students after completion of the third year of medical studies, with special emphasis on gender differences.

## 2. Materials and Methods

An open paper-based questionnaire was distributed among 200 randomly selected medical students at the Medical University of Graz, Austria. This public university runs a six-year medical program. All surveyed students had finished the third year of medical studies including education in the subject of Hygiene and Microbiology. All of them had already had patient contact. Confidentiality was protected by using only anonymized data, and the questionnaire had a cover to mask the questions. The questionnaire consisted of 20 single-choice questions (Table 1) and covered 3 areas.

1. Knowledge of hygiene guidelines regarding the daily clinical routine.
2. Adherence to hygiene guidelines [1] in the healthcare setting.
3. Satisfaction with current hygiene education, equipment, and quality standards in the framework of medical education.

All completed questionnaires without any contradiction (e.g., giving several answers to the same question or failure to complete the questionnaire) were included for evaluation. Completed questionnaires were coded. Descriptive measures were used to assess students' self-rated knowledge on and adherence to hygiene standards and if they were in favor of introducing mandatory quality standards, such as an operation theater license. Using chi-squared test, the above-mentioned variables were examined according to gender differences. The data were analyzed using SPSS 19.0 (SPSS Inc., Chicago, IL). *P* values ≤0.05 were considered statistically significant.

## 3. Results and Discussion

In the present study, 192 (96%) of 200 medical students returned questionnaires according to the inclusion criteria; 58% of the respondents were females and 42% were males. The mean age of the students was 23.5 years.

**Figure 1:**
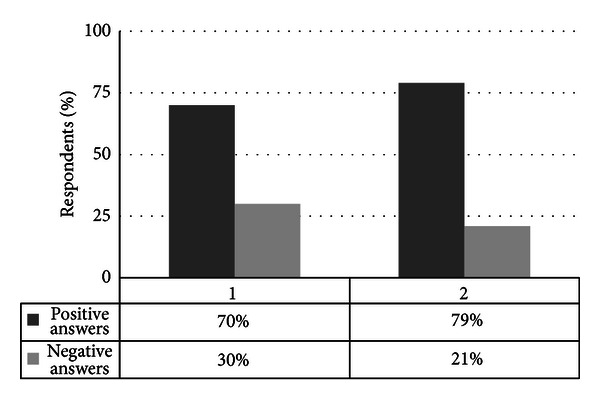
Self-assessment of knowledge and call for improved education. The *y*-axis shows the respondents in percentage. The *x*-axis gives the respondents' (1) self-rated knowledge on hygiene guidelines and (2) call for introducing mandatory quality standards in hygiene education, such as an operation theater license. In interpreting graph (1), knowledge assessed as “excellent” or “good” was classified as positive answer, whereas knowledge assessed as “satisfactory,” “sufficient,” and “insufficient” was classified as negative answer. Graph (2) shows the respondents favoring an operation theater license as positive answer versus those adverse to such a license.

Of the respondents, 70% judged their knowledge on hygiene guidelines as “excellent” or “good” and 49% of them reported adherence to hygiene guidelines (Figure 1). These results differ from those of Melenhorst et al. [12], who reported that only 6% of the participants judged their individual overall hygiene behavior as optimal in 90% of all patient contacts and 37% as optimal in 70% of all patient contacts. In general, a low level of correlation between self-assessment of knowledge and observed adherence to hygiene recommendations has been reported [12, 13]. Tibballs found that 73% of healthcare workers claimed adherence to hand hygiene guidelines, whereas the compliance rate observed was 10% [13]. This is in accordance with Snow et al., who showed that students report higher levels of hygiene adherence than actually observed [10]. Several other studies reported similar results among medical staff [14, 15]. Jenner et al. [16] concluded that surveys on adherence to hand hygiene are too unreliable and should be read with extreme caution. In the present study, the adherence rate may thus also have been overstated.

**Figure 2:**
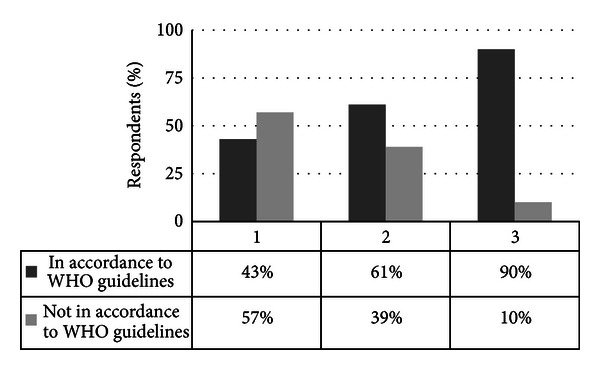
Compliance with WHO guidelines. The *y*-axis shows the respondents in percentage. The *x*-axis gives the respondents' (1) frequency of hygienic hand disinfection, (2) use of disposable gloves for blood sample collection, and (3) attitude towards “recapping” of needles. Each of the questions could be answered in accordance (dark bars) or not in accordance (light bars) with WHO guidelines.

Regarding adherence to hygiene guidelines, 57% of the respondents reported that they did not always follow hand disinfection recommendations and 39% did not wear gloves at all when collecting blood samples, while 90% of the students refrained from “recapping” of needles (Figure 2). Our findings show that the vast majority of medical students adhere to “no recapping” of needles, but more than one-third of the students do not wear gloves during collecting blood samples. Furthermore, only less than half of the respondents adhered to WHO guidelines on hygienic hand disinfection [1]. This low number is surprising since 92% of the respondents judged the disinfectant dispensers on the wards as absolutely sufficient and well accessible. In accordance with our data, previous studies have revealed that adherence to hand hygiene guidelines among healthcare workers is seldom over 40% [1, 2, 7, 8, 16–19]. Our data indicate a higher self-rated knowledge than adherence to hygiene guidelines among medical students (70% versus 49%). In accordance with the low data on active hygiene knowledge found in this study, Duroy and Le Coutour [19] reported that their study population was not aware of the difference between simple and antiseptic hand washing. Furthermore, Graf et al. [20] showed that only 21% of students were able to name all indications for proper hand hygiene correctly, and Mann and Wood [11] showed that 58% did not know the correct indications for using alcohol-based hand detergents. In contrast, Bittner et al. [14] showed that 90% of medical students reported adherence to WHO hand hygiene guidelines immediately after patient contact. Concerning the frequency of hygienic hand disinfection, the use of disposable gloves for blood sample collection, and “recapping” of needles, our results are in accordance with the findings of Edwards et al. [21] who described that 63% of the surveyed dental students performed hand hygiene procedures prior to and 60% after patient treatment. Over the years, numerous programs have been introduced in the hope of increasing the rates of compliance with hygiene standards. Unfortunately, adherence rates rarely exceed 40% prior to program implementation, and even after retraining, compliance is increased only temporarily [4, 14].

Asked about practices with direct patient contact, only 6% of the students did not shake hands when greeting their patients and 42% performed stethoscope disinfection after each patient contact. In contrast, 92% of the respondents judged the disinfectants provided on the wards as sufficient and 73% changed their white coats regularly. However, only 19% judged the number of white coats provided per student as sufficient. According to Wright et al. [22], stethoscopes are a potential source of nosocomial infection in the healthcare setting. In our study, WHO guidelines-compliant stethoscope disinfection following each patient contact was performed by only 42% of the participants. This is in accordance with Duroy and Le Coutour [19] who reported that 40% of students cleaned their stethoscope regularly. In contrast, Melenhorst et al. [12] reported that only 6% of the respondents performed stethoscope disinfection after each patient contact and only 5% changed white coats daily, which is in strong contrast to our findings. In this study, only 6% of the respondents refrained from shaking hands when greeting a patient. Although this is a characteristic feature of our culture, it is clearly against the WHO guidelines concerning hand hygiene [1].

Among all respondents, 74% reported that they had received a professional introduction to hygiene guidelines beyond their subject of Hygiene and Microbiology. This took place either in the framework of a teaching unit or a voluntary internship. Nevertheless, the majority of students (79%) favored the introduction of mandatory quality standards such as an operating theater license. This is in accordance with previous studies [12, 19] which demonstrated that most medical students are dissatisfied with hospital hygiene training and would consider it useful to receive more information on prevention of infections.

All variables examined showed no statistical significance between female and male students (*P* > 0.05). However, female students showed a better self-assessment regarding the knowledge of and adherence to hygiene guidelines in comparison to males. This supports the theory that female students may show better self-assessment than males regarding the knowledge of and adherence to hygiene guidelines. This is in accordance with the findings of recent studies [17, 23–25] that report a better hygiene-related behavior in women than men. However, two recent studies [7, 8] showed that male students practiced hand hygiene less often than their female counterparts only during the first observation period (prior to intervention). During the second observation period (after intervention), no significant differences were found. The major reason of lacking significances in the present study may be the conduction of the survey after completion of the subject of Hygiene and Microbiology; nevertheless, our findings indicate that women may adhere better to hygiene-related guidelines.

Limitations of our study are as follows.

1. The sample size (*n* = 200/192) was relatively small. For future studies, a higher number of participants is recommended.
2. Only 1 higher-education institution was sampled.
3. Self-reported hand hygiene adherence might be higher than adherence observed.
4. The individual number of clinical traineeships completed may affect the level of knowledge positively. The degree of experience in the clinical setting, for example, weeks of internships, was not recorded.

## 4. Conclusion

At medical universities, students are usually educated on hygiene-related guidelines only once during their medical education. This may result in serious deficiencies regarding the practical application of those guidelines. The current study showed the following.

1. The majority of the medical students surveyed reported adherence to hygiene-related guidelines in the clinical setting. Most of them judged their hygiene behavior as adequate.
2. Female students showed a better self-assessment regarding the knowledge of and adherence to hygiene guidelines in comparison to their male counterparts.
3. The majority of students demanded for further training and in-depth education on hygiene-related guidelines.

An increased emphasis on hygiene education, behavior, and assessment is needed for future physicians to gain optimal competence and to improve in-hospital patient safety. Ultimately, optimal medical education results in optimal patient care.

## Figures and Tables

**Table 1:** Distributed questionnaire. The survey was paper based and only single choice was accepted.

Question	Answer possibilities
How often do you disinfect your hands during a day in the clinical setting?	After every patient contact
	Before and after every patient contact
	Two-three times/day
	Never

How often do you disinfect the membrane of your stethoscope?	After every patient contact
	Before and after every patient contact
	Two-three times/day
	Never

How often do you disinfect your hands before or after blood sample collection?	After every patient contact
	Before and after every patient contact
	I wear gloves
	Never

How often do you wear gloves for blood sample collection?	Always
	Most of the time
	Never

How often do you perform “recapping” of needles?	Always
	Most of the time
	Never

How often do you change your white coat?	Regularly, at least every 1-2 weeks
	Sometimes, about every 1-2 months
	Only if the white coat is dirty
	I do not remember

How often do you wear your white coat during lunch break in the canteen?	Always
	Most of the time
	Never

Do you leave the operation wing with operation theater clothing?	Yes, always
	Sometimes
	Only if I go back in foreseeable time
	No, never

Assess your knowledge regarding hygiene guidelines in the OT (school marks)	Excellent
	Good
	Satisfactory
	Sufficient
	Not sufficient

How well do you know the hygiene guidelines at your university hospital?	Excellent
	Good
	Satisfactory
	Sufficient
	Not sufficient

Have you ever read and understood the hygiene guidelines?	Yes
	No
	I would like but I do not know where to find them

If you know the hygiene guidelines, would you have difficulties to state at least three guidelines?	Yes
	No

Would you favor the implementation of an obligatory operation theater license?	Yes
	No
	This would not solve the fundamental problem since adherence to hygiene guidelines decreases over time

Have you ever had a professional introduction to hygiene guidelines?	Yes, in the framework of a lecture or seminar
	Yes, by a doctor in the clinical setting
	Yes, in the framework of a clinical internship
	No

Do you think that the amount of disinfectant dispensers provided in the hospital is sufficient?	Yes
	No
	I always have a pocket dispenser with me

Do you wear bracelets, rings, or wristwatches in the hospital?	Yes, always
	Most of the time
	No, never

Do you sit on the hospital bed during patient contact?	Yes, always
	Rather often
	Sometimes
	Rather seldom
	No, never

Do you shake hands with the patient when greeting each other?	Yes, always
	Rather often
	Sometimes
	Rather seldom
	No, never

Do you think that there are sufficient numbers of white coats provided (one at a time) for students at your hospital?	Yes
	2-3 would be better
	At own disposal would be better

Have you ever had patient contact even if you were seriously ill, since you otherwise would not have fulfilled the requirements to take part in an exam?	Yes, repeatedly
	1-2 times during my medical studies so far
	No, never
